# OVR-GS: Open-Vocabulary 3D Object Removal via Semantic Gaussian Selection and Local Diffusion-Guided Completion

**DOI:** 10.3390/s26165258

**Published:** 2026-08-19

**Authors:** Yongpeng Ding, Feng Ouyang, Jiawei Fan, Ting Chen, Hongyan Xu

**Affiliations:** 1 College of Intelligent Technology, Tianfu College of Southwestern University of Finance and Economics, Mianyang 621000, China; dingyongpeng@tfswufe.edu.cn (Y.D.); ouyangfeng@tfswufe.edu.cn (F.O.); fanjiawei@tfswufe.edu.cn (J.F.); chengt@tfswufe.edu.cn (T.C.); 2 Mianyang Science and Technology City School-Enterprise Joint Application Technology Innovation Base for Low-Altitude Economy, Mianyang 621000, China

**Keywords:** 3D Gaussian splatting, open-vocabulary scene editing, score distillation sampling, language-guided perception, 3D inpainting, multi-view consistency

## Abstract

Camera-reconstructed 3D scenes often require offline visual cleanup before inspection, presentation, or reuse as renderable virtual-scene assets. Representative applications include removing temporary furniture, parked vehicles, equipment, signage, and other distracting or obsolete objects from reconstructed indoor and outdoor environments. Such editing requires not only accurate target localization across viewpoints but also plausible recovery of the previously occluded background. Existing methods often depend on manually specified masks or category-restricted detectors, while projection-based pipelines independently inpaint multiple views and subsequently refine the 3D representation, potentially introducing cross-view appearance and geometry inconsistencies. We present OVR-GS (Open-Vocabulary Removal in Gaussian Splatting), an instruction-driven object-removal framework for pre-trained 3D Gaussian Splatting (3DGS) scenes. Given a free-form instruction, a language parser generates target-oriented queries and a textual background-completion condition. Grounding DINO and the Segment Anything Model (SAM) produce multi-view candidate masks, which are filtered using Contrastive Language–Image Pre-training (CLIP). The proposed Semantic-Aware Gaussian Selector (SAGS) aggregates rendering-contribution-weighted mask evidence, groups spatially coherent candidates, and identifies the target Gaussian subset through rendered-cluster semantic verification. After removal, new Gaussians are initialized from boundary-adjacent primitives and interior samples and optimized locally using Score Distillation Sampling (SDS), while the original background remains fixed. On IMFine, SPIn-NeRF, and Inpaint360GS, OVR-GS achieves peak signal-to-noise ratio (PSNR) values of 19.78, 17.82, and 24.62 dB and Fréchet inception distance (FID) values of 142.30, 148.60, and 34.80, respectively. The results demonstrate the effectiveness of localized Gaussian optimization for instruction-driven cleanup of reconstructed environments before visual inspection, presentation, or reuse as renderable virtual-scene assets.

## 1. Introduction

Multi-view visual sensing systems support the reconstruction of photorealistic 3D environments for visual inspection, virtual-scene management, content presentation, and related downstream workflows. Recent advances in neural radiance fields (NeRF) [1,2] and 3D Gaussian Splatting (3DGS) [3] have substantially improved the quality and efficiency of reconstruction from calibrated camera observations. Nevertheless, reconstructed scenes may contain temporary, obsolete, or distracting objects that should be removed before the scene is inspected or reused. This operation requires both consistent localization of the target across viewpoints and plausible recovery of the geometry and appearance that were previously occluded.

A practical object-removal system must address three connected challenges. First, users should be able to specify arbitrary targets through natural-language descriptions rather than manually drawing masks in multiple views or relying on a fixed category set. Second, view-dependent and potentially noisy image observations must be converted into a reliable object-level selection within the 3D representation. Third, deleting the selected primitives leaves an unobserved region whose appearance and spatial structure must be completed without unnecessarily modifying the surrounding reconstruction.

Existing language-guided scene editors, Gaussian-based editing methods, and projection–inpainting–reconstruction pipelines address different parts of this problem. However, jointly supporting open-vocabulary target specification, robust Gaussian-level object selection, and spatially localized background completion remains challenging. In particular, independently inpainted views may provide inconsistent appearance or geometry, whereas scene-wide refinement updates substantially more parameters than necessary when the edit is confined to a small region.

To address these issues, we propose OVR-GS, an open-vocabulary object-removal framework for pre-trained 3DGS scenes. OVR-GS first converts a free-form instruction into target-localization queries and a separate background-completion condition. Multi-view proposals are then consolidated by the proposed Semantic-Aware Gaussian Selector (SAGS), which combines rendering-contribution evidence, spatial grouping, and rendered-cluster semantic verification to identify the target Gaussian subset. After removal, boundary-adjacent and interior Gaussians are initialized within the vacated region and refined using a frozen diffusion prior. The original background Gaussians remain fixed, so the editing process is restricted to newly introduced local primitives rather than re-optimizing the complete scene.

The main contributions of this work are summarized as follows:

- We present an open-vocabulary object-removal framework for pre-trained 3DGS scenes that integrates instruction-driven target specification, Gaussian-level object selection, and local background completion. The original scene representation outside the edited region remains fixed throughout the editing process.
- We introduce the Semantic-Aware Gaussian Selector, which converts noisy multi-view segmentation proposals into an object-level Gaussian selection through view-aware evidence accumulation, spatial clustering, and rendered-cluster semantic verification.
- We formulate background completion as a localized Gaussian optimization problem. Boundary-derived and interior Gaussian initialization provides a structured starting point, while diffusion-based score guidance and Gaussian regularization refine only the newly introduced primitives within the removed region.
- We evaluate OVR-GS on the IMFine, SPIn-NeRF, and Inpaint360GS benchmarks. The results demonstrate competitive reconstruction and perceptual quality across different scene layouts and camera trajectories while avoiding full-scene retraining during object removal.

## 2. Related Work

### 2.1. 3D Scene Representations for Editing

The choice of scene representation directly affects how accurately an edited region can be localized and how efficiently the resulting scene can be updated. Conventional explicit representations describe geometry through voxel grids [4], point clouds [5], or polygonal meshes [6]. These representations provide direct geometric access, but their memory requirements, discretization properties, or topology constraints can make them inconvenient for representing detailed scenes reconstructed from dense multi-view observations. Continuous implicit representations, including signed distance fields [7] and neural radiance fields (NeRF) [1], offer an alternative by encoding geometry or radiance as functions of spatial coordinates. Although NeRF supports high-quality novel-view synthesis, editing a localized object commonly requires modifying a scene-level neural representation through iterative optimization.

The introduction of 3D Gaussian Splatting (3DGS) [3] provides a different basis for scene editing. A 3DGS scene is represented by explicit Gaussian primitives whose positions, covariance, opacity, and appearance can be individually accessed, making localized manipulation more direct than in a fully implicit radiance field. Existing methods have exploited this structure for Gaussian grouping and text-guided editing [8,9]. Nevertheless, the representation itself does not determine which primitives correspond to a requested object, nor does it specify how the region exposed after removal should be reconstructed. Gaussian Grouping [8], for example, learns object-level identities within a reconstructed scene but still requires a scene-level grouping procedure before downstream editing can be performed.

Generative Gaussian representations constitute another related direction. DreamGaussian [10] and text-conditioned Gaussian generation methods [11] use diffusion priors to construct 3D content from textual or visual conditions. Their primary objective is to synthesize a new object or scene rather than to identify and remove a user-specified object from an existing reconstruction while preserving its surroundings. In contrast, the present work treats the pre-trained 3DGS scene as a fixed background representation and introduces additional mechanisms for Gaussian-level target identification and spatially localized completion.

### 2.2. Open-Vocabulary 3D Object Grounding

Open-vocabulary visual recognition enables objects to be specified by textual descriptions instead of a predefined category list. Vision–language pre-training, exemplified by CLIP [12], establishes a shared embedding space in which image regions can be compared with free-form text. Building on this capability, Grounding DINO, the Segment Anything Model, and their integrated pipelines [13,14,15] provide a practical route from a language query to candidate bounding boxes and pixel-level masks. These models operate on individual images, however, and therefore do not directly establish which elements of a reconstructed 3D representation belong to the queried object.

Text-conditioned 3D methods provide a complementary perspective. Text2NeRF [16] uses textual conditions to guide scene generation, while Instruct-NeRF2NeRF [17] propagates language-specified edits through a NeRF representation. These studies demonstrate the usefulness of natural language as an interface to 3D content, but their emphasis is scene generation or appearance modification rather than explicit correspondence between multi-view object observations and a subset of 3D Gaussian primitives.

The principal difficulty in Gaussian-level grounding is therefore not only obtaining a plausible mask in each view but also reconciling incomplete and potentially inconsistent masks across viewpoints. Occlusion, scale variation, false detections, and uncertain boundaries can cause a direct union of projected masks to include neighboring background primitives or omit parts of the target. OVR-GS addresses this intermediate problem by accumulating view-dependent mask evidence in Gaussian space, grouping spatially coherent candidates, and evaluating candidate groups through rendered-view semantic correspondence. The resulting selection process connects open-vocabulary 2D localization with object-level manipulation of an existing 3DGS scene.

### 2.3. Language-Guided 3D Scene Editing

Natural-language interfaces have been studied in several forms of embodied and visual interaction. SayCan [18], for example, connects language instructions with feasible actions in robotic environments, illustrating the broader role of semantic grounding in interactive systems. Within 3D scene editing, language guidance is more commonly combined with generative image priors. Instruct-NeRF2NeRF [17] repeatedly updates training views and optimizes a NeRF so that the requested edit becomes consistent within the reconstructed scene. For 3DGS, GaussCtrl [19] and EditSplat [20] improve view-consistent text-guided manipulation through multi-view conditioning and Gaussian-space optimization. These approaches primarily address appearance or content modification and generally retain an iterative scene-adaptation stage.

Object removal differs from general text-guided editing because deleting the selected representation leaves an unobserved region that must be completed. A widely used strategy first removes and inpaints the target in rendered or captured images and subsequently reconstructs or refines the 3D scene from the edited views. SPIn-NeRF [21], MVIP-NeRF [22], and GScream [23] follow variants of this projection–inpainting–reconstruction formulation. The approach benefits from established 2D inpainting models, but the generated views may contain differences in texture, boundaries, or inferred geometry that must be reconciled during 3D optimization.

Recent methods explicitly reduce this inconsistency. IMFine [24] incorporates geometry-guided multi-view refinement to coordinate inpainted observations, whereas Inpaint360GS [25] addresses object-aware completion under wide-baseline and full-rotation camera coverage. These developments show that both geometric correspondence and cross-view coordination are important for 3D inpainting. OVR-GS follows a related objective but adopts a different optimization scope: independently inpainted views are not used as fixed pseudo-targets for reconstructing the complete scene. Instead, the original background Gaussians are preserved, and completion is restricted to newly initialized primitives inside the removed region. This formulation separates local generative completion from the pre-trained representation of the unedited scene and avoids full-scene re-optimization during the editing stage.

## 3. Method

Let a pre-trained 3D Gaussian Splatting (3DGS) scene be represented as

(1) $$S={\left\{g_{i}=\left(\mu_{i},\Sigma_{i},\alpha_{i},c_{i}\right)\right\}}_{i=1}^{N},$$

where $\mu_{i}\in R^{3}$ and $\Sigma_{i}\in R^{3\times 3}$ denote the center and covariance of Gaussian $g_{i}$, $\alpha_{i}$ is its opacity, and $c_{i}$ contains its spherical-harmonic appearance coefficients. Given a free-form instruction I, our goal is to identify the Gaussian subset corresponding to the requested object, remove it, and complete the newly exposed region while preserving the original background representation.

The edited scene is expressed as

(2) $$S_{\mathrm{edit}}=\left(S\setminus G_{\mathrm{target}}\right)\cup G_{\mathrm{fill}}^{*},$$

where $G_{\mathrm{target}}$ is the selected target subset and $G_{\mathrm{fill}}^{*}$ is the locally optimized completion set. The parameters of $S\setminus G_{\mathrm{target}}$ remain fixed during completion.

**Figure 1:**
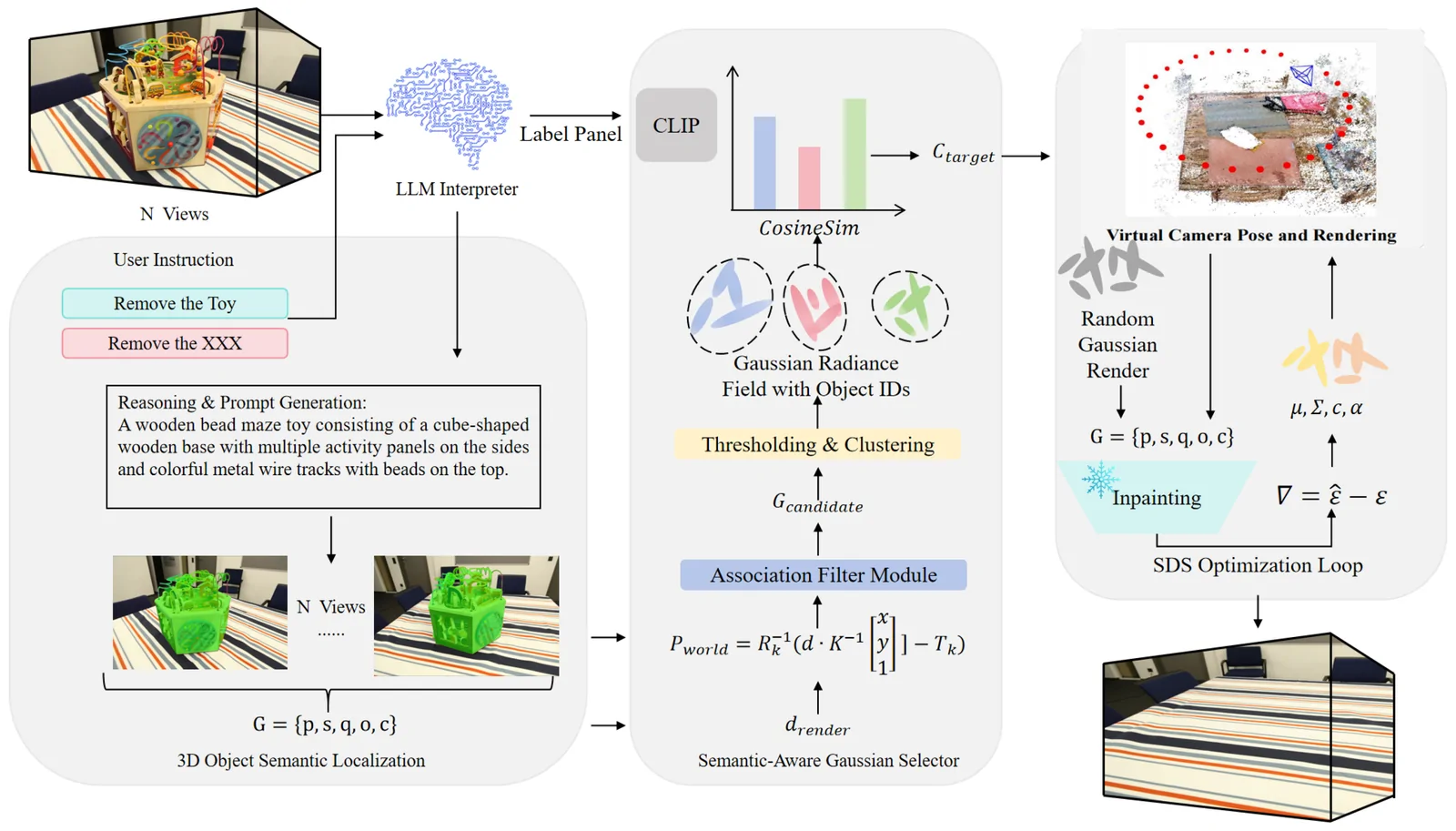
**Overview of the OVR-GS framework.** OVR-GS first generates and filters multi-view object masks from a free-form instruction. The Semantic-Aware Gaussian Selector (SAGS) then aggregates mask evidence in Gaussian space and identifies the target subset $G_{\mathrm{target}}$ through spatial clustering and semantic verification. After removal, new local Gaussians are initialized within the vacated region and optimized using score distillation sampling (SDS), while the original background Gaussians remain fixed.

As illustrated in Figure 1, OVR-GS consists of three main stages. Stage 1 converts the free-form editing instruction into target-oriented queries and a background-completion condition, and then generates and filters object proposals from multiple source views; this stage is detailed in Section 3.1. Stage 2 applies the Semantic-Aware Gaussian Selector to aggregate rendering-contribution evidence, construct spatial candidate clusters, and identify the target Gaussian subset through semantic verification, as described in Section 3.2. Stage 3 removes the selected subset and performs localized scene completion. The initialization of boundary-adjacent and interior Gaussians is presented in Section 3.3, while their context-preserved SDS-guided optimization is described in Section 3.4.

### 3.1. Language-Conditioned Multi-View Proposals

The first stage converts an unrestricted editing instruction into multi-view image evidence that can subsequently be associated with the 3D Gaussian representation.

#### 3.1.1. Instruction Decomposition

We use Qwen2.5-7B-Instruct as the language parser $P_{\mathrm{LLM}}$. Given an instruction I, the parser separates target localization from background completion:

(3) $$\left(Q_{\mathrm{target}},y_{\mathrm{bg}}\right)=P_{\mathrm{LLM}}\left(I\right),$$

where

(4) $$Q_{\mathrm{target}}=\left\{q_{1},q_{2},\ldots ,q_{K}\right\},\;K\leq 3,$$

contains one canonical target name and at most two synonymous or attribute-enhanced localization expressions. The second output, $y_{\mathrm{bg}}$, is a concise description of the surface or scene content expected after removal and is used only during local completion.

The parser uses deterministic decoding with temperature 0, top-p=1.0, and a maximum output length of 256 tokens. It returns a JSON record containing the fields target_name, localization_queries, and background_condition. The parsed result is cached once per instruction and reused throughout localization and repeated runs.

For example, the instruction “*Remove the visible cables and complete the wall behind them*” is converted into the canonical target “*cable*”; the queries {“*cable*”, “*wire*”, and “*thin hanging line*”}; and the background condition “*a continuous wall surface matching the surrounding color and texture*”. Separating the two outputs prevents the removed-object description from being directly reused as the desired completion.

#### 3.1.2. Grounded Segmentation over Source Views

We use the calibrated training cameras as source viewpoints,

(5) $$V_{\mathrm{src}}=\left\{v_{1},v_{2},\ldots ,v_{M}\right\}.$$

Rendering the pre-trained scene from viewpoint $v_{m}$ produces an RGB image $I_{m}$ and depth map $D_{m}$:

(6) $$\left(I_{m},D_{m}\right)=R\left(S,v_{m}\right),$$

where R denotes the differentiable Gaussian renderer.

For each rendered image, Grounding DINO [13] predicts text-conditioned bounding boxes:

(7) $$B_{m}=\mathrm{GroundingDINO}\left(I_{m},Q_{\mathrm{target}}\right).$$

Each proposal $B_{m,j}\in B_{m}$ is used as a box prompt for HQ-SAM [14,15]:

(8) $$M_{m,j}=\mathrm{HQSAM}\left(I_{m},B_{m,j}\right),\;M_{m,j}\in {\left\{0,1\right\}}^{H\times W}.$$

These masks are treated as view-dependent hypotheses rather than final object labels because a proposal can contain background regions or only a visible fragment of the target.

#### 3.1.3. Query-Aware Proposal Filtering

We suppress semantically weak proposals using frozen CLIP encoders [12]. Let $I_{m,j}^{\mathrm{crop}}$ be the crop enclosed by $B_{m,j}$. Its semantic confidence is

(9) $$\begin{array}{c} s_{m,j}^{\mathrm{clip}}=\max_{q_{k}\in Q_{\mathrm{target}}}\frac{E_{\mathrm{img}}{\left(I_{m,j}^{\mathrm{crop}}\right)}^{⊤}E_{\mathrm{txt}}\left(q_{k}\right)}{{∥E_{\mathrm{img}}\left(I_{m,j}^{\mathrm{crop}}\right)∥}_{2}{∥E_{\mathrm{txt}}\left(q_{k}\right)∥}_{2}}. \end{array}$$

A proposal is retained when $s_{m,j}^{\mathrm{clip}}\geq \tau_{\mathrm{clip}}$. The accepted set is

(10) $${\hat{M}}_{m}=\left\{M_{m,j}\;|\;s_{m,j}^{\mathrm{clip}}\geq \tau_{\mathrm{clip}}\right\}.$$

When several accepted proposals overlap, their pixel-wise union forms the foreground support in view *m*:

(11) $${\hat{M}}_{m}\left(u\right)=\max_{M_{m,j}\in {\hat{M}}_{m}}M_{m,j}\left(u\right).$$

The collection $\hat{M}={\left\{{\hat{M}}_{m}\right\}}_{m=1}^{M}$ is passed to SAGS as multi-view semantic evidence.

### 3.2. Semantic-Aware Gaussian Selector

A 2D mask does not directly identify the Gaussians belonging to the corresponding object. Multiple depth-ordered Gaussians can contribute to one pixel, and the visible object region can vary substantially across views. SAGS therefore aggregates foreground evidence according to the actual alpha-compositing contributions and then performs spatial grouping and semantic verification.

SAGS operates directly on the Gaussian primitives provided by the pre-trained reconstruction and therefore assumes that the target object and its surrounding background are represented by sufficiently dense and spatially localized Gaussians. This assumption is important because the subsequent evidence scores are derived from the alpha-compositing contributions of the existing primitives. Reconstruction artifacts, including floaters, holes, duplicated surfaces, or under-reconstructed regions, can consequently bias the accumulated foreground evidence before spatial clustering and semantic verification. SAGS can suppress inconsistent mask observations, but it cannot recover target geometry that is absent from the underlying 3DGS representation.

#### 3.2.1. Rendering-Contribution Evidence Aggregation

Let $\Omega_{m}$ denote the pixel domain of source view $v_{m}$. For a Gaussian $g_{i}$ contributing to pixel u, its rasterization weight is

(12) $$w_{m,u,i}=T_{m,u,i}\alpha_{m,u,i},$$

where $\alpha_{m,u,i}$ includes the learned opacity and the projected 2D Gaussian footprint, and

(13) $$T_{m,u,i}=\prod_{j<i}\left(1-\alpha_{m,u,j}\right)$$

is the accumulated transmittance before $g_{i}$ along the corresponding ray.

For each Gaussian, we accumulate the foreground-supported contribution and its total visible contribution:

(14) $$\begin{array}{cc} A_{i} & =\sum_{m=1}^{M}\sum_{u\in \Omega_{m}}w_{m,u,i}{\hat{M}}_{m}\left(u\right), \end{array}$$

(15) $$\begin{array}{cc} Z_{i} & =\sum_{m=1}^{M}\sum_{u\in \Omega_{m}}w_{m,u,i}. \end{array}$$

The normalized evidence score is

(16) $$e_{i}=\frac{A_{i}}{Z_{i}+\xi },\;e_{i}\in \left[0,1\right],$$

where $\xi =10^{-8}$ prevents numerical instability. Thus, $e_{i}$ measures the fraction of the visible rendering contribution of Gaussian $g_{i}$ supported by the accepted foreground masks. Gaussians with $Z_{i}<10^{-6}$ are treated as insufficiently observed and are not used for candidate construction.

#### 3.2.2. Spatial Candidate Grouping

The preliminary candidate set is

(17) $$G_{\mathrm{cand}}=\left\{g_{i}\in S\;|\;e_{i}\geq \tau_{\mathrm{conf}}\right\}.$$

Before clustering, Gaussian centers are normalized by subtracting the scene centroid and dividing by the maximum center-to-centroid distance. The candidate centers are then grouped using DBSCAN [26]:

(18) $$C=\left\{C_{1},C_{2},\ldots ,C_{L}\right\}=\mathrm{DBSCAN}\left(\left\{{\tilde{\mu }}_{i}\;|\;g_{i}\in G_{\mathrm{cand}}\right\};\varepsilon ,n_{\min}\right),$$

where ${\tilde{\mu }}_{i}$ is the normalized center, ε is the neighborhood radius, and $n_{\min}$ is the minimum number of points required to form a dense region. DBSCAN is used only to organize coherent candidates and remove isolated outliers; the target identity is determined by the subsequent semantic verification.

#### 3.2.3. Rendered-Cluster Semantic Verification

For every cluster $C_{l}$, we choose the source view in which the cluster has the largest visible projected area:

(19) $$v_{l}^{*}=\underset{v_{m}\in V_{\mathrm{src}}}{\arg\max}\;a_{\mathrm{proj}}\left(C_{l},v_{m}\right),$$

where $a_{\mathrm{proj}}\left(C_{l},v_{m}\right)$ denotes the visible image-space area of the cluster. The Gaussians in $C_{l}$ are rendered in isolation:

(20) $$I_{l}^{\mathrm{iso}}=R\left(G\left(C_{l}\right),v_{l}^{*}\right),$$

with

(21) $$G\left(C_{l}\right)=\left\{g_{i}\in G_{\mathrm{cand}}\;|\;{\tilde{\mu }}_{i}\in C_{l}\right\}.$$

The cluster-level semantic score is

(22) $$\begin{array}{c} s_{l}^{\mathrm{sem}}=\max_{q_{k}\in Q_{\mathrm{target}}}\frac{E_{\mathrm{img}}{\left(I_{l}^{\mathrm{iso}}\right)}^{⊤}E_{\mathrm{txt}}\left(q_{k}\right)}{{∥E_{\mathrm{img}}\left(I_{l}^{\mathrm{iso}}\right)∥}_{2}{∥E_{\mathrm{txt}}\left(q_{k}\right)∥}_{2}}. \end{array}$$

The selected cluster and target subset are

(23) $$l^{*}=\underset{1\leq l\leq L}{\arg\max}\;s_{l}^{\mathrm{sem}},$$

(24) $$G_{\mathrm{target}}=G\left(C_{l^{*}}\right).$$

The maximization in Equation (23) returns only one spatial cluster. Consequently, the current implementation is designed primarily for removing a single dominant target instance. When an instruction refers to multiple spatially disconnected instances of the same category, such as “remove all chairs”, several clusters may exhibit high semantic compatibility, but only the highest-scoring cluster is included in $G_{\mathrm{target}}$. The remaining valid instances are therefore retained. Supporting such collective instructions would require a calibrated multi-cluster selection rule rather than the current single-cluster maximization.

### 3.3. Local Completion Initialization

Removing $G_{\mathrm{target}}$ produces the fixed background scene

(25) $$S_{\mathrm{bg}}=S\setminus G_{\mathrm{target}}.$$

The removed primitives define both the spatial extent of the missing region and the camera neighborhood used for completion.

#### 3.3.1. Spatial Envelope of the Removed Region

We first construct a convex envelope from the centers of the removed Gaussians:

(26) $$H_{\mathrm{void}}=\mathrm{ConvHull}\left(\left\{\mu_{i}\;|\;g_{i}\in G_{\mathrm{target}}\right\}\right).$$

The hull is used only as a compact initialization region and is not assumed to reproduce the exact object geometry. For a nearly planar or otherwise degenerate target whose normalized hull volume is below $10^{-6}$, we instead use a padded oriented bounding box with a minimum thickness of 0.02 times its longest side. Let $o_{\mathrm{void}}$ and $V(H_{\mathrm{void}})$ denote the resulting envelope centroid and volume, respectively, and let $d_{\mathrm{void}}$ be the diagonal length of its axis-aligned bounding box.

#### 3.3.2. Coverage-Constrained Virtual Cameras

We construct virtual cameras centered at $o_{\mathrm{void}}$ and restrict their azimuth and elevation ranges to those supported by the calibrated source cameras. For wide-baseline, 180°, and 360° scenes, we sample $N_{\mathrm{virt}}=12$ views; for scenes with approximately 90° camera coverage, we use four views within the observed range.

The initial trajectory radius is set to the mean source-camera distance to $o_{\mathrm{void}}$ and is then adjusted until the projected void occupies between 1% and 50% of the rendered image. This constraint prevents the missing region from becoming too small to provide useful guidance or too large for reliable diffusion completion. The virtual cameras use the intrinsics of the nearest source camera in pose space.

#### 3.3.3. Boundary-and-Interior Gaussian Seeding

Purely random initialization does not exploit the observed surfaces around the removed region. We therefore combine boundary-derived clones with interior samples. The boundary-neighbor set is

(27) $$G_{\mathrm{bd}}=\left\{g_{i}\in S_{\mathrm{bg}}\;|\;d\left(\mu_{i},\partial H_{\mathrm{void}}\right)\leq \delta \right\},\;\delta =0.05d_{\mathrm{void}}.$$

Gaussians in $G_{\mathrm{bd}}$ are cloned and displaced toward $o_{\mathrm{void}}$ by $0.02d_{\mathrm{void}}$. The clones retain the covariance and appearance coefficients of their parent primitives.

Boundary cloning alone can leave a large central region under-represented. We estimate a local Gaussian density $\rho_{\mathrm{bd}}$ in the shell surrounding $\partial H_{\mathrm{void}}$ and set

(28) $$N_{\mathrm{int}}=\mathrm{clip}\left(\left\lceil \rho_{\mathrm{bd}}V\left(H_{\mathrm{void}}\right)\right\rceil ,1000,\;20,000\right).$$

We uniformly sample $N_{\mathrm{int}}$ centers in the envelope. Their appearance coefficients are copied from the nearest boundary primitive, and their opacity is initialized to $\alpha_{0}=0.05$. The initial isotropic scale is

(29) $$s_{0}=\kappa {\left(\frac{V(H_{\mathrm{void}})}{N_{\mathrm{int}}}\right)}^{1/3},\;\kappa =0.8.$$

The initial completion set is

(30) $$G_{\mathrm{fill}}^{0}=G_{\mathrm{bd}}^{\mathrm{clone}}\cup G_{\mathrm{int}}.$$

The optimization scene is

(31) $$S_{\mathrm{opt}}=S_{\mathrm{bg}}^{\mathrm{frozen}}\cup G_{\mathrm{fill}}^{\mathrm{learnable}},$$

and only the parameters of $G_{\mathrm{fill}}^{\mathrm{learnable}}$ participate in optimization.

### 3.4. Context-Preserved SDS-Guided Local Completion

The initialized Gaussians provide spatial support but do not yet encode the geometry and appearance of the newly exposed background. We refine them using Score Distillation Sampling (SDS) from a frozen diffusion inpainting model. All virtual views render and update the same learnable Gaussian set, so their guidance signals are coupled implicitly through one shared 3D representation rather than applied to independent 2D completion variables.

We do not impose an explicit cross-view photometric consistency loss inside the projected void. The original observations do not contain the object-free appearance of the occluded region, and such a loss would therefore require either independently generated view-specific pseudo-targets or estimated correspondences across an initially unknown completion surface. Independently generated pseudo-targets may disagree in texture, boundary placement, and inferred geometry, while unreliable correspondences could impose incorrect supervision during the early stages of Gaussian optimization. We therefore use the shared Gaussian representation as an implicit multi-view coupling mechanism and restrict explicit RGB and depth preservation to the observed context surrounding the void.

#### 3.4.1. Inpainting-Conditioned Latent Guidance

Let

(32) $$\theta_{\mathrm{fill}}=\left\{\mu_{i},\Sigma_{i},\alpha_{i},c_{i}\;|\;g_{i}\in G_{\mathrm{fill}}\right\}$$

denote the learnable local parameters. For a virtual view $\tilde{v}\in V_{\mathrm{virt}}$, we render the current composite RGB image and depth map:

(33) $$\left(x_{\tilde{v}},D_{\tilde{v}}\right)=R\left(S_{\mathrm{bg}}^{\mathrm{frozen}}\cup G_{\mathrm{fill}}\left(\theta_{\mathrm{fill}}\right),\tilde{v}\right).$$

We also render the frozen background alone,

(34) $$\left(x_{\tilde{v}}^{\mathrm{bg}},D_{\tilde{v}}^{\mathrm{bg}}\right)=R\left(S_{\mathrm{bg}}^{\mathrm{frozen}},\tilde{v}\right),$$

and project $H_{\mathrm{void}}$ to obtain a binary completion mask $m_{\tilde{v}}$. The masked conditioning image is

(35) $$x_{\tilde{v}}^{\mathrm{mask}}=\left(1-m_{\tilde{v}}\right)⊙x_{\tilde{v}}^{\mathrm{bg}}.$$

Thus, the diffusion model receives the complete surrounding scene and an explicit mask, rather than an isolated crop of the new primitives.

We use the frozen Stable Diffusion 2 Inpainting checkpoint (stabilityai/stable-diffusion-2-inpainting) as the score prior. No diffusion-model parameter is updated. The current composite and masked conditioning image are encoded using the frozen VAE:

(36) $$z=E_{\mathrm{vae}}\left(x_{\tilde{v}}\right),\;z_{\mathrm{mask}}=E_{\mathrm{vae}}\left(x_{\tilde{v}}^{\mathrm{mask}}\right).$$

At timestep *t*, noise ϵ∼N(0,I) is added as

(37) $$z_{t}=\sqrt{{\bar{\alpha }}_{t}}\;z+\sqrt{1-{\bar{\alpha }}_{t}}\;\epsilon ,$$

where ${\bar{\alpha }}_{t}$ follows the pre-trained diffusion schedule. The downsampled mask, masked-image latent, noised latent, and text condition are passed to the frozen inpainting UNet.

Following SDS [27] and subsequent score-distillation formulations [28], the surrogate gradient is

(38) $$\begin{array}{c} \nabla_{\theta_{\mathrm{fill}}}L_{\mathrm{SDS}}=E_{\tilde{v},t,\epsilon }\left[w\left(t\right)\left({\hat{\epsilon }}_{\phi }\left(z_{t};z_{\mathrm{mask}},m_{\tilde{v}},y_{\mathrm{bg}},t\right)-\epsilon \right)\frac{\partial z}{\partial x_{\tilde{v}}}\frac{\partial x_{\tilde{v}}}{\partial \theta_{\mathrm{fill}}}\right], \end{array}$$

where

(39) $$w\left(t\right)=1-{\bar{\alpha }}_{t}.$$

The gradient is taken only with respect to $\theta_{\mathrm{fill}}$; all original background parameters are excluded from the optimizer.

#### 3.4.2. Boundary and Context Preservation

Freezing the background prevents parameter updates to the original scene, but it does not prevent newly introduced Gaussians from projecting beyond the intended completion region. We therefore reduce their influence near the edit boundary by applying a local context-preservation constraint within a narrow image-space band. Let

(40) $$\Omega_{\mathrm{bd}}^{\tilde{v}}=\mathrm{Dilate}\left(m_{\tilde{v}},r_{\mathrm{bd}}\right)\setminus m_{\tilde{v}}$$

denote the ring immediately outside the projected completion mask. We preserve the frozen RGB and depth values in this observed region using

(41) $$\begin{array}{cc} L_{\mathrm{ctx}}\; & =\frac{1}{|\Omega_{\mathrm{bd}}^{\tilde{v}}|}\sum_{u\in \Omega_{\mathrm{bd}}^{\tilde{v}}}{∥x_{\tilde{v}}\left(u\right)-x_{\tilde{v}}^{\mathrm{bg}}\left(u\right)∥}_{1}\\ \; & \;+\lambda_{D}\frac{1}{|\Omega_{\mathrm{bd}}^{\tilde{v}}|}\sum_{u\in \Omega_{\mathrm{bd}}^{\tilde{v}}}\left|D_{\tilde{v}}\left(u\right)-D_{\tilde{v}}^{\mathrm{bg}}\left(u\right)\right|. \end{array}$$

This term preserves the observed appearance and depth continuity near the edit boundary while leaving the masked interior to be determined by the diffusion prior. The narrow-band formulation is used because reliable RGB and depth references are available immediately outside the projected void, whereas imposing such supervision inside the void would constrain genuinely unobserved content. However, $L_{\mathrm{ctx}}$ is a local image-space constraint rather than a global support constraint on $G_{\mathrm{fill}}$. It does not explicitly penalize the rendering contribution of a newly initialized Gaussian at pixels farther outside the boundary band, nor does it guarantee that the same Gaussian remains inside the projected completion region under every possible camera viewpoint.

#### 3.4.3. Opacity and Density Regularization

SDS guidance can activate unnecessary primitives or leave Gaussians in weakly opaque states. We therefore use a binary-opacity term

(42) $$L_{\mathrm{bin}}=\frac{1}{|G_{\mathrm{fill}}|}\sum_{g_{i}\in G_{\mathrm{fill}}}\alpha_{i}\left(1-\alpha_{i}\right),$$

and an opacity sparsity term

(43) $$L_{\mathrm{sparse}}=\frac{1}{|G_{\mathrm{fill}}|}\sum_{g_{i}\in G_{\mathrm{fill}}}\left|\alpha_{i}\right|.$$

The complete local objective is

(44) $$L_{\mathrm{total}}=L_{\mathrm{SDS}}+\lambda_{\mathrm{ctx}}L_{\mathrm{ctx}}+\lambda_{\mathrm{bin}}L_{\mathrm{bin}}+\lambda_{\mathrm{sparse}}L_{\mathrm{sparse}}.$$

After optimization,

(45) $$G_{\mathrm{fill}}^{*}=G_{\mathrm{fill}}\left(\theta_{\mathrm{fill}}^{*}\right),$$

and the edited scene follows Equation (2).

## 4. Experiments

### 4.1. Experimental Setup

#### 4.1.1. Datasets

We evaluate OVR-GS on three benchmarks with different scene layouts, camera trajectories, and evaluation protocols. IMFine [24] contains 20 indoor and outdoor scenes captured under diverse camera trajectories, including front-facing, 90°, 180°, and 360° settings. It also covers different surface configurations such as single-plane, multi-plane, curved, and irregular surfaces. Each scene provides 175 frames with camera poses and object masks, making it suitable for evaluating object removal under wide viewpoint changes and complex geometry. SPIn-NeRF [21] consists of 10 real-world forward-facing scenes with paired object-present and object-free captures. Following the standard protocol, 60 images are used for reconstruction and 40 held-out object-free views are used for quantitative evaluation. Inpaint360GS [25] focuses on 360° capture scenarios, which provide a challenging setting for assessing multi-view consistency under full-rotation viewpoint coverage.

#### 4.1.2. Evaluation Metrics

We adopt four standard metrics to evaluate object erasure and scene completion quality: peak signal-to-noise ratio (PSNR) [29], structural similarity index measure (SSIM), learned perceptual image patch similarity (LPIPS) [30], and Fréchet inception distance (FID) [31]. PSNR measures pixel-level fidelity between the edited rendering and the object-free ground truth, while SSIM evaluates structural similarity. LPIPS measures perceptual similarity using deep features, and FID evaluates distribution-level realism. Higher PSNR and SSIM values indicate better reconstruction fidelity, whereas lower LPIPS and FID values indicate better perceptual quality and distributional realism.

For quantitative comparisons with prior methods, metrics are computed on held-out object-free views following the corresponding benchmark protocols. For datasets without pixel-aligned object-free ground truth, we provide qualitative comparisons instead. Unless otherwise specified, all ablation results in this study are computed using the same full-image evaluation protocol as the main comparison on the SPIn-NeRF benchmark.

#### 4.1.3. Implementation Details

We follow the official reconstruction and evaluation protocol of each benchmark. SPIn-NeRF uses 60 object-present views for reconstruction and 40 held-out object-free views for quantitative evaluation. For IMFine and Inpaint360GS, we adopt their official scene splits and evaluation views. Each input scene is first reconstructed using the standard 3D Gaussian Splatting pipeline. During editing, all original background Gaussians remain fixed, and only newly initialized local completion Gaussians are optimized.

We use Qwen2.5-7B-Instruct as the instruction parser. It is executed with greedy decoding, temperature 0, top-p=1.0, and a maximum output length of 256 tokens. For every editing instruction, the parser returns one canonical target name, at most three localization queries, and one background-completion condition in a fixed JSON format. The parsed outputs are cached and reused throughout all experiments.

For object localization, we use Grounding DINO with the Swin-T OGC checkpoint. The box and text thresholds are 0.35 and 0.25, respectively. Each accepted box is passed to HQ-SAM with the ViT-H checkpoint. HQ-SAM is used with its default inference configuration and without task-specific fine-tuning. Candidate crops are encoded using the frozen CLIP ViT-B/32 model and retained with $\tau_{\mathrm{clip}}=0.25$.

SAGS accumulates mask evidence using the per-pixel alpha-compositing weights exported by the differentiable Gaussian rasterizer. Gaussians with normalized evidence $e_{i}\geq 0.50$ are retained as candidates. Before clustering, scene coordinates are normalized to a unit-radius coordinate system centered at the scene centroid. DBSCAN uses ε=0.10 and $n_{\min}=5$. The spatial cluster with the highest isolated-rendering CLIP similarity is selected as the target Gaussian subset.

For wide-baseline, 180°, and 360° scenes, we sample $N_{\mathrm{virt}}=12$ virtual views. For scenes with approximately 90° camera coverage, four virtual views are sampled within the observed camera range. The trajectory radius is adaptively adjusted so that the projected removal region occupies approximately 1–50% of the rendered image. Boundary-adjacent Gaussians are selected within $0.05d_{\mathrm{void}}$ of the completion-volume boundary and are displaced toward the void centroid by $0.02d_{\mathrm{void}}$. Interior Gaussian density is matched to the local boundary density, with the number of interior primitives clipped to the range [1000, 20,000]. Their initial opacity is 0.05, and the scale coefficient is set to κ=0.8.

We use the frozen Stable Diffusion 2 Inpainting model (stabilityai/stable-diffusion-2-inpainting) as the score prior. The model receives the complete composite rendering, the projected completion mask, and the generated background condition. SDS-guided local optimization is performed for 500 iterations at 512×512 resolution. Timesteps are uniformly sampled from [0.02,0.98], with a classifier-free guidance scale of 100 and $w\left(t\right)=1-{\bar{\alpha }}_{t}$.

The learnable local Gaussians are optimized using Adam with $\beta_{1}=0.9$, $\beta_{2}=0.99$, $\epsilon =10^{-8}$, and a common learning rate of $3\times 10^{-5}$. The loss weights are $\lambda_{\mathrm{bd}}=1.0$, $\lambda_{\mathrm{bin}}=0.01$, and $\lambda_{\mathrm{sparse}}=0.001$. Densification, opacity reset, and pruning are disabled during completion. All settings are fixed across scenes unless explicitly varied in the sensitivity analysis.

The reported defaults were selected according to controlled ablation and sensitivity analyses and were then kept fixed across all three benchmarks. Categorical design choices were assessed by comparing semantic prompting and CLIP verification strategies and by comparing random, boundary-only, and boundary-plus-interior Gaussian initialization. Continuous settings were selected according to the joint PSNR, SSIM, and LPIPS results, with runtime used as an additional criterion when the quality difference was marginal. In particular, 500 SDS iterations provide the best measured quality before over-optimization becomes apparent, while 12 virtual views provide nearly saturated quality at lower cost than 16 views. The selected CLIP threshold and DBSCAN radius balance false-proposal admission against target fragmentation or merging. No scene-specific parameter adjustment is performed in the reported experiments.

All experiments are conducted on a single NVIDIA H800 GPU. The reported editing time excludes the one-time reconstruction of the initial 3DGS scene. Under the reported configuration, target proposal generation and SAGS selection require approximately 3 min, while local SDS-guided completion requires approximately 55 min, resulting in an average total editing time of approximately 58 min per scene.

### 4.2. Comparison with State-of-the-Art Methods

Table 1 compares OVR-GS with recent 3D object removal and scene-inpainting methods on the IMFine, SPIn-NeRF, and Inpaint360GS benchmarks. The compared methods are divided into 2D projection models, which edit rendered views before reconstructing or updating the 3D scene, and 3D representation models, which perform removal and completion directly within the underlying scene representation. In addition to established baselines, we include the recent Gaussian-based methods SplatFill and GOR-IS and evaluate them under the same benchmark protocol.

Overall, OVR-GS achieves the best result in eight of the nine reported metric columns and ranks second in the remaining column. On IMFine, OVR-GS obtains 19.78 dB PSNR, 0.2670 LPIPS, and 142.30 FID, outperforming the strongest previous results by 0.11 dB, 0.0015, and 7.22 points, respectively. The consistent improvements across pixel-level fidelity, perceptual similarity, and distributional quality indicate that the proposed completion strategy remains effective across the diverse camera trajectories represented in IMFine.

On SPIn-NeRF, OVR-GS achieves the highest PSNR of 17.82 dB and the lowest FID of 148.60. Compared with the recent SplatFill baseline, OVR-GS improves PSNR by 0.17 dB, reduces LPIPS from 0.4012 to 0.3950, and lowers FID by 4.25 points. It also consistently outperforms GOR-IS across all three metrics. GScream obtains the lowest LPIPS of 0.3931, while OVR-GS ranks second with 0.3950; however, OVR-GS provides substantially higher PSNR and lower FID, suggesting a more balanced reconstruction in terms of fidelity and perceptual quality.

The advantage is also maintained on the full-rotation Inpaint360GS benchmark. OVR-GS achieves 24.62 dB PSNR, 0.1275 LPIPS, and 34.80 FID. Compared with SplatFill, which provides the second-best PSNR, OVR-GS improves PSNR by 0.14 dB. Compared with Inpaint360GS, which provides the second-best LPIPS and FID, OVR-GS reduces LPIPS by 0.0025 and FID by 1.13 points. These results demonstrate that OVR-GS can preserve rendering quality and perceptual coherence under wide-baseline and full-rotation camera trajectories, while avoiding the degradation commonly observed when independently edited 2D views are projected back into a 3D scene.

### 4.3. Per-Scene Breakdown Analysis

To provide deeper insight into OVR-GS’s robustness across diverse scene types, Table 2 presents per-scene results on the SPIn-NeRF benchmark, comparing our method against the strongest baseline (IMFine). Table 2 shows that OVR-GS consistently improves over IMFine across all 10 SPIn-NeRF scenes and all three metrics. On average, OVR-GS improves PSNR from 17.58 dB to 17.82 dB, increases SSIM from 0.733 to 0.754, and reduces LPIPS from 0.4513 to 0.3950, corresponding to a 12.5% relative reduction in perceptual error. The largest PSNR gain appears on *Bag* (+0.84 dB), suggesting that the proposed SDS-driven local completion is particularly effective when the removed region exposes relatively uniform background content. Larger gains are also observed on *Chair* (+0.31 dB), *Hydrant* (+0.29 dB), and *Toy* (+0.29 dB), where accurate target selection and coherent local inpainting help reduce residual artifacts. The smallest improvement occurs on *Cone* (+0.03 dB), indicating that simple objects on clean backgrounds leave limited room for further PSNR improvement. Overall, the per-scene results indicate that OVR-GS provides consistent but scene-dependent gains over the strongest baseline, with more pronounced advantages in cases where semantic target localization and 3D-native completion are more critical.

### 4.4. Qualitative Comparison

**Figure 2:**
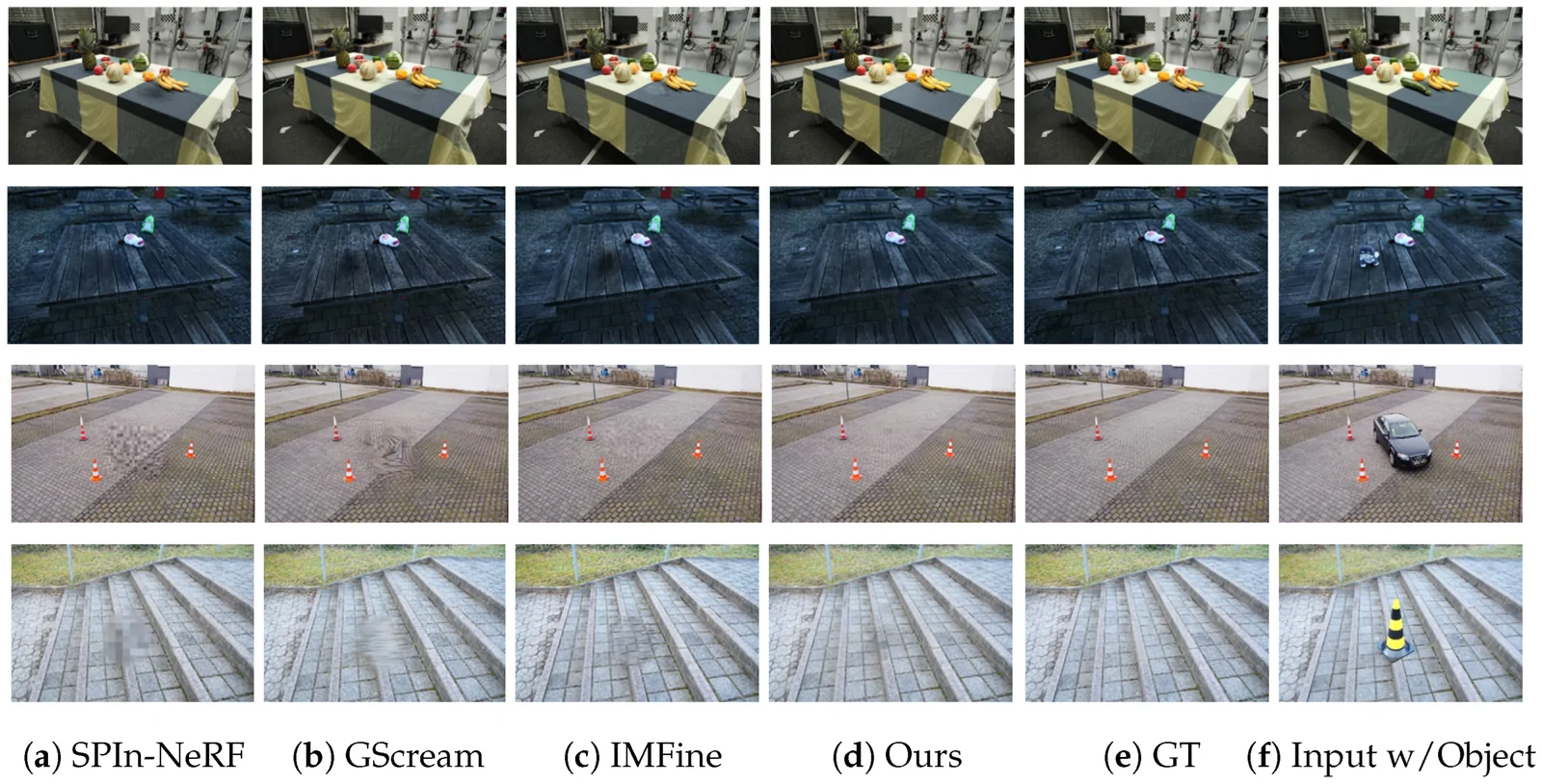
**Qualitative comparison on representative scenes.** Columns (**a**–**c**) show the results of SPIn-NeRF [21], GScream [23], and IMFine [24], respectively; column (**d**) shows the OVR-GS result; column (**e**) is the object-free reference; and column (**f**) is the input containing the target object. The examples mainly represent conditions in which the targets are visually distinguishable and the surrounding regions provide sufficient contextual evidence. Under these conditions, OVR-GS generally produces fewer residual fragments and less visible boundary discontinuity than the compared methods.

Figure 2 presents visual comparisons between OVR-GS and three representative baselines, namely SPIn-NeRF, GScream, and IMFine. The selected examples mainly represent standard operating conditions in which the target object is visually distinguishable from its surroundings and the adjacent region provides sufficient appearance evidence for completion. Under these conditions, OVR-GS generally reduces visible seams, blurred regions, and residual object fragments compared with the representative baselines.

Accordingly, Figure 2 should be interpreted as illustrating representative successful cases rather than as a complete evaluation of the operating boundary of the method. More difficult conditions, including reflective or transparent appearance, severe occlusion, thin or fragmented structures, ambiguous same-category targets, and weakly textured backgrounds, are discussed in Section 5.3. These conditions may affect target selection, completion-region construction, or diffusion-guided optimization in different ways.

### 4.5. Multi-View Geometric Inspection

**Figure 3:**
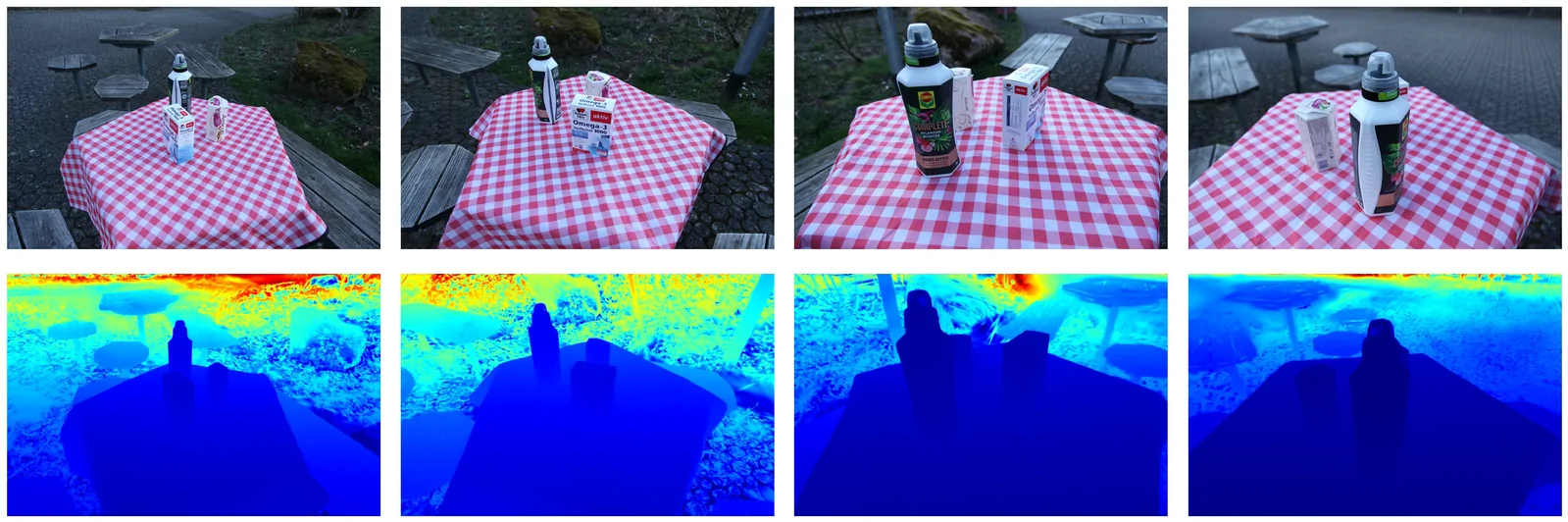
**Multi-view inspection of the completed appearance and geometry.** The top row shows RGB renderings of the edited scene from four held-out camera viewpoints, and the bottom row shows the corresponding rendered depth maps. These viewpoints are not used during local SDS optimization. The visualization is intended to assess viewpoint-dependent geometric continuity rather than absolute metric-depth accuracy.

To examine whether the locally completed region remains stable under viewpoint changes, Figure 3 presents the edited RGB renderings and corresponding depth maps from four held-out camera poses. These poses span the available evaluation trajectory and are excluded from the virtual views used during SDS optimization. The completed region remains spatially attached to the surrounding surface across the evaluated views, without obvious view-dependent displacement or large floating structures. Minor depth variation can still be observed near uncertain boundaries, particularly when the removed object originally occludes weakly textured or poorly reconstructed surfaces.

### 4.6. Runtime and Efficiency Analysis

Table 3 compares the editing time and peak GPU memory of OVR-GS with representative baselines under a unified evaluation protocol. All methods were evaluated on the same NVIDIA H800 GPU using the same input scenes, image resolution, and timing scope. Each method was executed using its official implementation and recommended configuration. The reported runtime excludes the one-time initial scene reconstruction and covers target selection, object removal, and the subsequent inpainting or refinement stage. Each measurement is averaged over three timed runs after one warm-up run.

OVR-GS does not eliminate optimization. Instead, it keeps the original background Gaussians fixed and restricts parameter updates to the newly initialized inpainting Gaussians. Consequently, the method does not require full-scene re-optimization after target removal. Target proposal generation and SAGS-based Gaussian selection require approximately 3 min, while local SDS-guided completion requires approximately 55 min. The complete editing pipeline therefore takes approximately 58 min and reaches a peak GPU memory usage of 16 GB.

Under the unified protocol, OVR-GS requires less total editing time than GaussianEditor, SPIn-NeRF, and IMFine while also exhibiting the lowest peak GPU memory usage among the evaluated methods. The stage-wise breakdown further shows that the main computational cost of OVR-GS arises from diffusion-guided local completion rather than target selection. These results indicate that restricting optimization to the removed region reduces the cost associated with updating the complete scene representation, although the local SDS refinement stage remains computationally demanding.

### 4.7. Ablation Study

#### 4.7.1. Component-Wise Analysis

We extend the component-wise ablation study from SPIn-NeRF to all three benchmarks to examine whether the principal modules remain effective under different scene configurations and camera trajectories. As reported in Table 4, removing any component degrades PSNR, LPIPS, and FID on SPIn-NeRF, IMFine, and Inpaint360GS, demonstrating that the improvements are not specific to a single dataset.

SDS-guided completion provides the largest and most consistent contribution. Removing this stage decreases PSNR by 1.74, 1.18, and 2.17 dB on SPIn-NeRF, IMFine, and Inpaint360GS, respectively, while increasing LPIPS by 0.1174, 0.0750, and 0.0705. Without generative completion, the system primarily propagates observed boundary appearance and cannot reliably reconstruct the geometry and texture of the previously occluded region. Removing SAGS also causes substantial degradation, reducing PSNR by 1.29 dB on SPIn-NeRF, 0.66 dB on IMFine, and 0.77 dB on Inpaint360GS. This confirms the importance of consolidating noisy view-level masks through rendering-contribution evidence, spatial grouping, and semantic verification before Gaussian removal.

LLM-guided parsing provides smaller but stable improvements across the three benchmarks, with PSNR reductions of 0.56, 0.33, and 0.52 dB when it is removed. Its contribution arises from generating more reliable target-localization expressions and separating the target description from the background-completion condition. The effect of boundary-and-interior initialization is more dependent on camera coverage. Its removal causes relatively limited PSNR decreases of 0.27 and 0.13 dB on SPIn-NeRF and IMFine, but a considerably larger decrease of 1.42 dB on Inpaint360GS. The full-rotation setting exposes the initialized Gaussian distribution from a wider range of viewpoints, making insufficient interior support, floating primitives, and boundary discontinuities more visible. Overall, the cross-dataset results show that the four components are complementary, although their relative contributions vary with viewpoint coverage, scene geometry, and target visibility.

**Figure 4:**
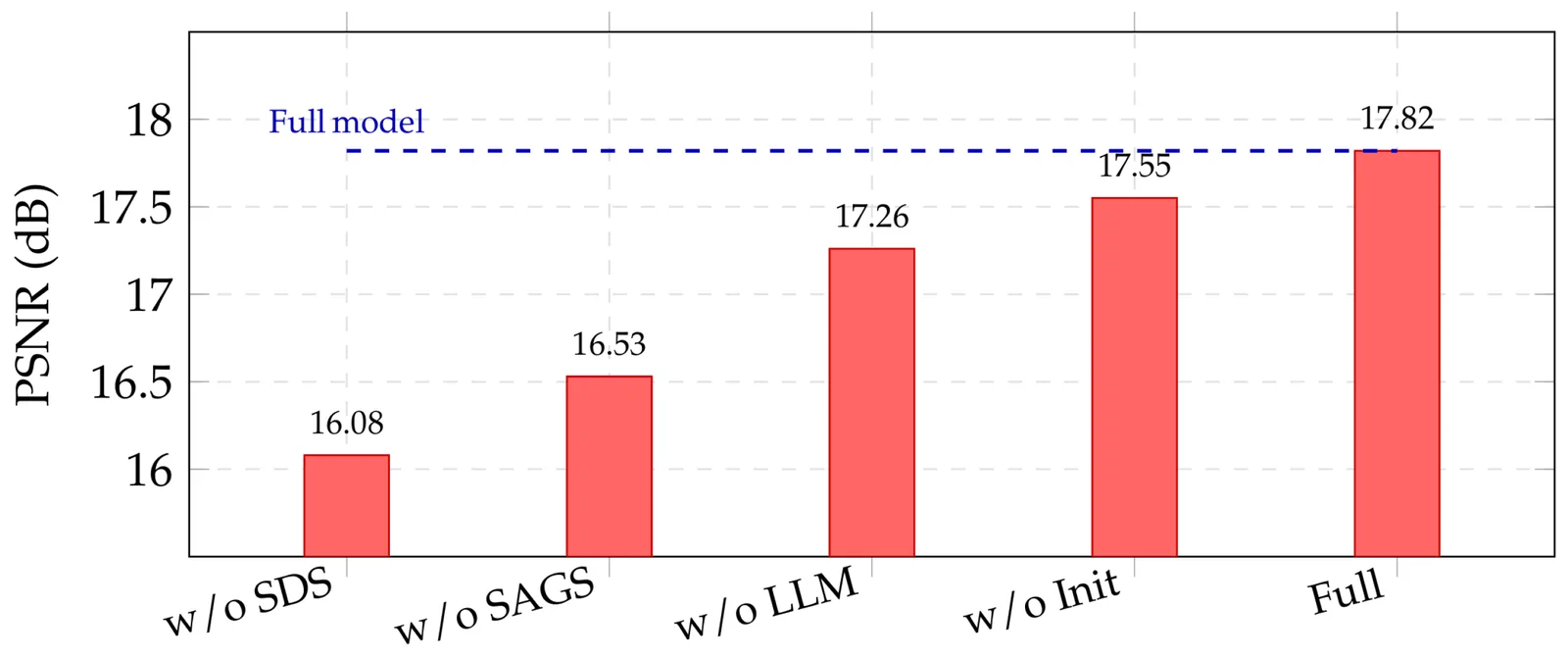
**Component-wise ablation measured by peak signal-to-noise ratio (PSNR).** Each bar shows the PSNR obtained when the corresponding component is removed. The dashed line marks the full-model performance of 17.82 dB. Score distillation sampling (SDS)-based inpainting contributes the largest margin (+1.74 dB), followed by the Semantic-Aware Gaussian Selector (SAGS; +1.29 dB), large language model (LLM) parsing (+0.56 dB), and boundary initialization (+0.27 dB).

Figure 4 visualizes the incremental PSNR contribution of each component, ordered by impact magnitude.

#### 4.7.2. Impact of SDS Optimization Iterations

**Figure 5:**
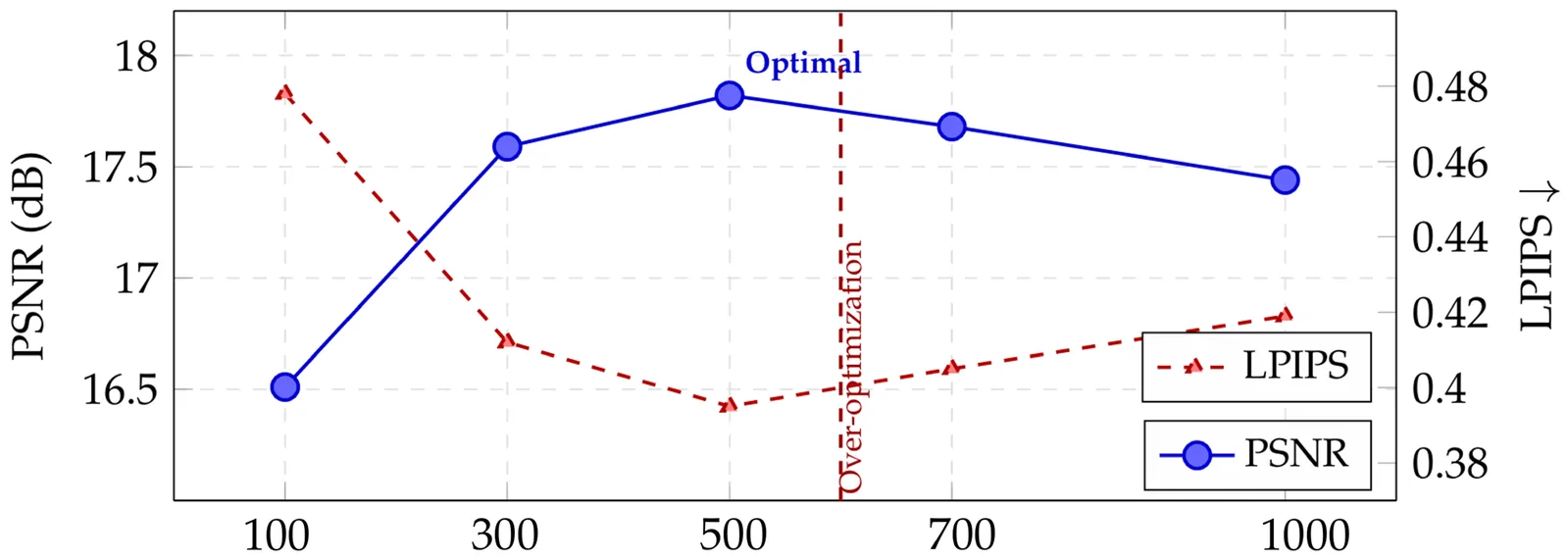
**Score distillation sampling (SDS) optimization iterations versus quality metrics.** Both peak signal-to-noise ratio (PSNR; solid line, left axis) and learned perceptual image patch similarity (LPIPS; dashed line, right axis) reach their optimum at 500 iterations. The downward arrow indicates that a lower LPIPS value is better. Beyond this point, over-optimization introduces floater artifacts and degrades both metrics. The red vertical dashed line marks the onset of diminishing returns.

Table 5 examines how the number of SDS optimization iterations affects inpainting quality. The quality curve exhibits a clear unimodal profile. Under-optimization (100–300 iterations) leaves the void only partially filled, producing blurry, incomplete geometry, whereas over-optimization (>700 iterations) drives the diffusion prior to injecting high-frequency “floater” artifacts and color over-saturation [27]. The sweet spot at 500 iterations attains the best trade-off across all three metrics, simultaneously maximizing PSNR/SSIM and minimizing LPIPS. This trend is visualized in Figure 5.

#### 4.7.3. Effectiveness of SAGS Target Selection

Table 6 isolates the contribution of CLIP verification within SAGS, averaged over 50 language instructions.

Full SAGS attains a commanding F1-score of 0.89, outperforming 2D mask voting (+12 pts) and random cluster selection (+27 pts). The decisive factor is precision, which leaps by +26.5% (0.86 vs. 0.68): the CLIP render-and-verify step acts as a semantic gatekeeper that effectively reduces false positives in cluttered scenes, exactly the failure mode that derails geometry-only selectors. Coupled with a near-saturated recall of 0.93, this confirms that SAGS captures almost the entire target while admitting almost no background contamination, which is precisely the property required for clean, hole-free removal.

#### 4.7.4. Ablation on SAGS Sub-Components

Table 7 further disentangles the contributions of LLM prompting and CLIP verification within SAGS, measured by their impact on final rendering quality. The two semantic ingredients of SAGS reinforce each other. Adding CLIP verification on top of a fixed prompt lifts PSNR by 0.44 dB (17.18 vs. 16.74) and trims LPIPS by 0.030, suppressing false positives that would otherwise carve unrecoverable holes into the background. Replacing the fixed prompt with LLM-generated queries contributes a further 0.64 dB (17.82 vs. 17.18) by enabling fine-grained semantic disambiguation among look-alike candidates. The two modules are clearly synergistic, since combining them recovers a full 1.61 dB over the random-cluster baseline, and without CLIP verification, boundary artifacts appear in 68% of the test cases.

#### 4.7.5. Impact of Gaussian Initialization Strategy

Table 8 compares three initialization approaches for the inpainting Gaussians. Random initialization lacks boundary priors and scatters primitives across free space, producing fragmented structures and a sluggish SDS convergence. Boundary-only copying sharpens the edges but starves the interior, leaving the core of the void under-covered. Our combined strategy preserves seamless edge transitions via cloned boundary Gaussians while supplying sufficient interior primitives for SDS to refine, lifting PSNR by 1.43 dB and reducing LPIPS by 18.9% relative to random initialization.

#### 4.7.6. Hyperparameter Sensitivity Analysis

Table 9 analyzes the sensitivity of OVR-GS to key hyperparameters. OVR-GS is remarkably robust to moderate hyperparameter variations, with every configuration remaining within 0.5 dB of the optimal PSNR, a strong indication that our gains stem from the framework design rather than from delicate parameter tuning. A lower $\tau_{clip}$ (0.20) admits false positives (−0.41 dB), while a higher value (0.30) over-prunes valid detections (−0.24 dB). A tight DBSCAN ϵ (0.05) fragments the target (−0.49 dB), whereas a loose ϵ (0.15) merges it with adjacent structures (−0.30 dB). Increasing the virtual views from 12 to 16 yields a negligible change (−0.04 dB) while incurring roughly 33% longer runtime, confirming that 12 views already saturate coverage.

### 4.8. Visualization of SAGS Process

**Figure 6:**
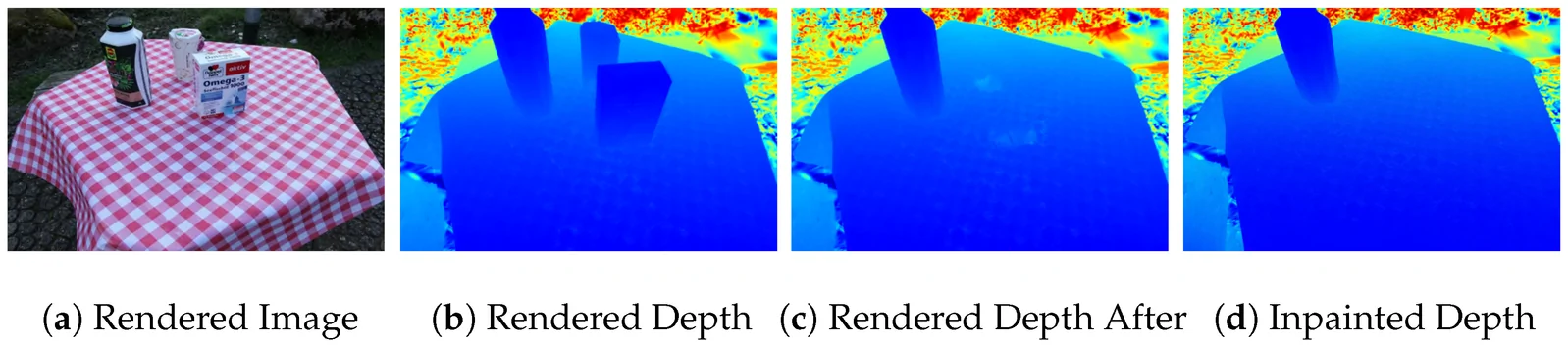
**Visualization of the Semantic-Aware Gaussian Selector (SAGS) and depth-inpainting process.** (**a**) The rendered red–green–blue (RGB) image of the target scene. (**b**) The rendered depth map before object removal. (**c**) The depth map after removing the target Gaussian subset $G_{\mathrm{target}}$, revealing the geometric void. (**d**) The completed depth map after the void is filled by the proposed generative three-dimensional inpainting procedure.

Figure 6 visualizes the intermediate steps of the SAGS module, demonstrating how multi-view 2D masks are progressively refined into a precise 3D Gaussian selection.

### 4.9. SDS Optimization Progression

The evolution of inpainting quality during SDS optimization is illustrated quantitatively in Figure 5 (Section 4.7.2). The inpainting region evolves from a sparse, low-opacity initialization to photorealistic completion within approximately 500 iterations. At early stages (100–300 iterations), the diffusion prior primarily establishes coarse geometry and dominant color patterns; between 300 and 500 iterations, fine-grained texture and boundary details progressively emerge. Beyond 700 iterations, over-optimization leads to the well-known “floater” artifacts and color over-saturation, validating our default setting of 500 iterations.

## 5. Discussion

### 5.1. Methodological Insights

OVR-GS addresses object removal through two closely connected operations: Gaussian-level target selection and localized generative completion. For target selection, open-vocabulary masks obtained from individual views may contain false positives, incomplete boundaries, and viewpoint-dependent omissions. Directly combining the primitives associated with these masks may therefore remove nearby background structures or retain parts of the target. SAGS mitigates this problem by aggregating normalized rendering-contribution evidence across multiple views, organizing high-confidence candidates into spatially coherent clusters, and verifying each cluster according to the semantic similarity between its isolated rendering and the target description. The ablation results indicate that this combination of multi-view evidence, spatial grouping, and semantic verification is more reliable than view-level mask voting or geometry-only selection.

After target removal, OVR-GS represents the missing region using a shared set of newly initialized Gaussians. Unlike pipelines that independently inpaint multiple images and subsequently reconstruct the scene, all virtual views in OVR-GS update the same Gaussian positions, covariances, opacities, and appearance coefficients. Guidance sampled from different viewpoints is therefore coupled through a common 3D representation. Boundary-adjacent Gaussians provide appearance and structural cues from the observed surroundings, while interior samples provide sufficient capacity to represent the previously occluded region. Binary-opacity and sparsity regularization further suppress semi-transparent and redundant primitives.

This shared representation improves multi-view coordination but does not constitute an explicit geometric-consistency guarantee. The SDS gradient is still evaluated separately for each sampled virtual view, and different viewpoints may favor different plausible completion modes. An explicit photometric reprojection loss is also difficult to construct because no object-free RGB observation exists inside the removed region. Using independently generated 2D inpainting results as supervision would reintroduce the view-dependent pseudo-target inconsistencies that the localized 3D formulation is intended to avoid. OVR-GS therefore adopts a practical trade-off: it preserves the observed background, constrains the completion near its boundary, and uses a shared Gaussian representation to coordinate the unknown interior implicitly.

### 5.2. Practical Scope

OVR-GS is primarily intended for offline visual editing of static scenes reconstructed as 3D Gaussian Splatting representations. Representative applications include removing temporary furniture, vehicles, equipment, signage, or other unwanted objects before a reconstructed environment is inspected, presented, or reused as a renderable virtual-scene asset. The open-vocabulary interface reduces the need for category-specific detectors and manually annotated multi-view masks, while localized optimization preserves the original background Gaussians outside the edited region.

The method assumes that a calibrated and pre-trained 3DGS scene is already available. It should also not be interpreted as eliminating optimization altogether. In the reported implementation, target selection requires approximately three minutes, while SDS-guided local completion accounts for most of the total editing time of approximately 58 min. OVR-GS avoids full-scene retraining by restricting updates to newly introduced local primitives, but it is not currently suitable for real-time or continuously changing environments.

The output of OVR-GS is an edited 3DGS representation rather than a complete digital-twin or simulation asset. Applications requiring polygonal topology, collision geometry, physical material parameters, semantic metadata, or platform-specific interfaces would require additional representation conversion and task-specific validation. The claims of this work are therefore limited to visual editing and completion of pre-trained 3DGS scenes.

### 5.3. Limitations and Future Work

The first group of limitations concerns target identification. Thin, transparent, reflective, or heavily occluded objects may occupy only a small number of pixels, change substantially in appearance across viewpoints, or be represented by sparse and fragmented Gaussians. These conditions reduce both view-level segmentation quality and Gaussian-level association reliability. Ambiguous instructions may also correspond to several plausible objects, while the current implementation retains only the spatial cluster with the highest semantic score. Consequently, collective commands such as “remove all chairs” and fragmented multi-component targets are not fully supported. These difficulties may be further amplified by errors in the pre-trained 3DGS reconstruction: floaters, duplicated surfaces, holes, and under-reconstructed regions can distort rendering-contribution evidence and propagate into the estimated completion region. Interactive clarification, uncertainty-aware evidence aggregation, and instruction-conditioned multi-cluster selection are promising directions for improving robustness.

A controlled quantitative comparison across reflective surfaces, thin structures, and different occlusion levels is not provided in the current study. The adopted benchmarks do not contain standardized annotations or severity levels for these properties, and assigning such categories after observing the results would introduce subjective post hoc labeling. Future benchmarks with explicit challenge annotations and sufficient samples at different severity levels would enable a more rigorous measurement of performance degradation under these conditions. In addition, although all evaluated benchmarks contain real-camera observations acquired in physical environments, the current study does not include an independently collected author-captured test set. Further evaluation on newly captured real-world scenes, including qualitative assessment when object-free ground truth is unavailable, would provide additional evidence of practical generalization beyond established public benchmarks.

The second group of limitations concerns completion fidelity. The quality of the generated region depends on the semantic and geometric priors of the frozen diffusion model. Prolonged or unstable SDS optimization may produce over-saturated colors, floaters, or semantically inconsistent details, particularly when the missing surface is weakly constrained by the visible surroundings. Moreover, the completion envelope is estimated from the distribution of the removed Gaussians and may only approximate the actual occluded surface for thin, planar, or irregular targets. Newly initialized Gaussians may also develop projected support outside the intended completion region, leading to color leakage or floating density from unobserved viewpoints. More stable score-distillation objectives [36,37], surface-aware completion domains, three-dimensional support constraints, and confidence-weighted reprojection or depth-consistency terms could improve geometric reliability and cross-view coherence.

Finally, the current framework remains computationally expensive and is limited to single-instance removal in static scenes. Faster generative guidance based on consistency models [38], cached diffusion features, reduced-view optimization, or lightweight distillation could substantially reduce the local completion cost. Extending SAGS to instruction-aware multi-cluster selection would support multi-instance and multi-object editing, while mechanisms for resolving overlapping completion regions and object-removal order would be required when selected objects occlude one another. Integration with dynamic 4D Gaussian representations [39] is another important direction for time-varying scenes.

## 6. Conclusions

We presented OVR-GS, an open-vocabulary object-removal framework for pre-trained 3D Gaussian Splatting scenes. The proposed Semantic-Aware Gaussian Selector converts multi-view segmentation evidence into a Gaussian-level target subset through normalized evidence aggregation, spatial clustering, and rendered-cluster semantic verification. After target removal, OVR-GS initializes new Gaussians within the vacated region and optimizes only these local primitives under guidance from a frozen diffusion prior, while keeping the original background representation fixed. The method therefore avoids full-scene retraining without eliminating the local optimization required for background completion. Experiments on SPIn-NeRF, IMFine, and Inpaint360GS show that OVR-GS achieves competitive reconstruction and perceptual quality across different scene layouts and camera trajectories. The ablation results further indicate that semantic Gaussian selection, boundary-aware initialization, and SDS-guided local completion each contribute positively to the final performance. Runtime analysis shows that removing full-scene re-optimization reduces the overall editing cost, although the local diffusion-guided completion stage remains the main computational bottleneck. Experiments on IMFine, SPIn-NeRF, and Inpaint360GS show that OVR-GS achieves PSNR values of 19.78, 17.82, and 24.62 dB and FID values of 142.30, 148.60, and 34.80, respectively. The cross-dataset ablation further shows that removing SDS-guided completion decreases PSNR by 1.18–2.17 dB, confirming its consistent contribution to restoration quality. Runtime analysis also indicates that restricting optimization to local Gaussians avoids full-scene re-optimization, although diffusion-guided completion remains the main computational bottleneck. The current framework is primarily designed for static scenes and single-object removal. Its performance may degrade for thin, transparent, reflective, or heavily occluded objects, where both multi-view segmentation and Gaussian-level association become less reliable. The method also inherits errors from the pre-trained 3DGS reconstruction; severe floaters, holes, or under-reconstructed regions may degrade both Gaussian-level target selection and the subsequent construction of the completion region. Future work will investigate more stable and efficient score-distillation objectives, multi-component and multi-object selection, and extensions to dynamic 4D Gaussian representations.

## Figures and Tables

**Table 1:** **Quantitative comparison on the IMFine, SPIn-NeRF, and Inpaint360GS benchmarks.** Bold indicates the best result, underline indicates the second-best result.

Method	IMFine			SPIn-NeRF			Inpaint360GS		
	PSNR ↑	LPIPS ↓	FID ↓	PSNR ↑	LPIPS ↓	FID ↓	PSNR ↑	LPIPS ↓	FID ↓
*2D Projection Models*									
GaussianEditor [32]	15.71	0.6163	375.03	14.41	0.6247	343.16	–	–	–
GScream [23]	17.18	0.4431	290.63	16.96	**0.3931**	154.71	20.95	0.2715	206.25
SPIn-NeRF [21]	18.75	0.3519	206.43	17.47	0.5740	239.99	19.71	0.5002	229.95
MVIP-NeRF [22]	18.63	0.4332	278.99	17.67	0.5070	255.51	–	–	–
AuraFusion360 [33]	–	–	–	–	–	–	23.15	0.1915	47.71
GauGroup [8]	–	–	–	–	–	–	23.20	0.1770	65.87
*3D Representation Models*									
SplatFill [34]	19.54	0.2738	156.42	17.65	0.4012	152.85	24.48	0.1335	36.92
GOR-IS [35]	19.41	0.2815	159.78	17.53	0.4085	155.40	24.25	0.1392	39.15
Inpaint360GS [25]	–	–	–	–	–	–	24.40	0.1300	35.93
IMFine [24]	19.67	0.2685	149.52	17.58	0.4513	154.34	–	–	–
**OVR-GS (Ours)**	**19.78**	**0.2670**	**142.30**	**17.82**	0.3950	**148.60**	**24.62**	**0.1275**	**34.80**

*Note:* PSNR denotes peak signal-to-noise ratio; LPIPS denotes learned perceptual image patch similarity; and FID denotes Fréchet inception distance. 2D and 3D denote two-dimensional and three-dimensional, respectively. ↑ indicates that a higher value is better, whereas ↓ indicates that a lower value is better. “–” indicates that the corresponding method did not report results under the same benchmark and evaluation protocol.

**Table 2:** **Per-scene comparison on the SPIn-NeRF benchmark.** Results are reported for IMFine and OVR-GS across ten scenes, and the average row is shown in bold for readability.

Scene	IMFine			OVR-GS (Ours)		
	PSNR ↑	SSIM ↑	LPIPS ↓	PSNR ↑	SSIM ↑	LPIPS ↓
Statue	18.25	0.763	0.4218	18.32	0.779	0.3694
Trash Can	17.92	0.740	0.4382	18.11	0.760	0.3817
Bike	16.86	0.699	0.4896	17.03	0.720	0.4304
Bench	17.43	0.724	0.4614	17.57	0.744	0.4023
Cone	18.70	0.788	0.3981	18.73	0.802	0.3548
Hydrant	17.09	0.713	0.4757	17.38	0.733	0.4162
Chair	18.34	0.770	0.4123	18.65	0.787	0.3605
Pillow	17.57	0.736	0.4548	17.64	0.751	0.3976
Toy	16.73	0.692	0.4974	17.02	0.714	0.4389
Bag	16.91	0.705	0.4637	17.75	0.750	0.3982
**Average**	**17.58**	**0.733**	**0.4513**	**17.82**	**0.754**	**0.3950**

*Note:* PSNR denotes peak signal-to-noise ratio; SSIM denotes structural similarity index measure; and LPIPS denotes learned perceptual image patch similarity. ↑ indicates that a higher value is better, whereas ↓ indicates that a lower value is better; the arrows indicate the preferred metric direction rather than the row-sorting order.

**Table 3:** Runtime comparison under a unified evaluation protocol. All methods were evaluated using their official implementations on the same NVIDIA H800 GPU, with the same input scenes, image resolution, and timing scope. Each method used its officially recommended scene representation and configuration. Runtime excludes the one-time initial scene reconstruction and includes target selection, object removal, and subsequent inpainting or refinement. The reported values are averaged over three timed runs after one warm-up run. Bold formatting is used to highlight the proposed method.

Method	Full-Scene Retraining (min)	Target Selection (min)	Local Inpainting Refinement (min)	Total (min)	GPU Memory (GB)
GaussianEditor	45	2 (manual)	15	62	18
SPIn-NeRF	60	5	20	85	22
IMFine	50	8	25	83	20
**OVR-GS (Ours)**	**0**	**3**	**55**	**58**	**16**

**Table 4:** **Cross-dataset component-wise ablation of OVR-GS.** Each variant is evaluated on the SPIn-NeRF, IMFine, and Inpaint360GS benchmarks. The best result for each metric on each dataset is shown in bold.

Variant	SPIn-NeRF			IMFine			Inpaint360GS		
	PSNR ↑	LPIPS ↓	FID ↓	PSNR ↑	LPIPS ↓	FID ↓	PSNR ↑	LPIPS ↓	FID ↓
w/o LLM-guided parsing	17.26	0.4271	162.40	19.45	0.2850	155.20	24.10	0.1380	48.50
w/o SAGS module	16.53	0.4783	185.60	19.12	0.3050	168.40	23.85	0.1520	58.20
w/o boundary-consistent initialization	17.55	0.4108	158.30	19.65	0.2750	149.80	23.20	0.1650	65.40
w/o SDS-guided completion	16.08	0.5124	215.80	18.60	0.3420	195.30	22.45	0.1980	72.60
**Full model (Ours)**	**17.82**	**0.3950**	**148.60**	**19.78**	**0.2670**	**142.30**	**24.62**	**0.1275**	**34.80**

*Note:* LLM denotes large language model; SAGS denotes Semantic-Aware Gaussian Selector; SDS denotes score distillation sampling; and “w/o” denotes “without”. PSNR denotes peak signal-to-noise ratio; LPIPS denotes learned perceptual image patch similarity; and FID denotes Fréchet inception distance. ↑ and ↓ indicate that higher and lower values are preferred, respectively.

**Table 5:** **Effect of the number of score-distillation optimization iterations on inpainting quality.** The best overall balance is obtained at 500 iterations and is shown in bold.

SDS Iterations	PSNR ↑	SSIM ↑	LPIPS ↓
100	16.51	0.701	0.4780
300	17.59	0.745	0.4120
**500**	**17.82**	**0.754**	**0.3950**
700	17.68	0.749	0.4050
1000	17.44	0.738	0.4190

*Note:* SDS denotes score distillation sampling; PSNR denotes peak signal-to-noise ratio; SSIM denotes structural similarity index measure; and LPIPS denotes learned perceptual image patch similarity. ↑ indicates that a higher value is better, whereas ↓ indicates that a lower value is better; the arrows do not indicate the ordering of the tested iteration settings.

**Table 6:** **Effectiveness of contrastive language–image pre-training verification in the Semantic-Aware Gaussian Selector for target selection.** Results are averaged over 50 instructions, and the best values are shown in bold.

Selection Strategy	Recall ↑	Precision ↑	F1-Score ↑
2D Mask Voting Only	0.89	0.68	0.77
Random Cluster Selection	0.72	0.55	0.62
**Full SAGS (Ours)**	**0.93**	**0.86**	**0.89**

*Note:* SAGS denotes Semantic-Aware Gaussian Selector; and 2D denotes two-dimensional. The F1-score is the harmonic mean of precision and recall. ↑ indicates that a higher value is better and does not indicate the row-sorting order.

**Table 7:** **Ablation of semantic prompting and contrastive language–image verification within the Semantic-Aware Gaussian Selector.** Final rendering metrics are reported, and the best results are shown in bold.

SAGS Configuration	PSNR ↑	SSIM ↑	LPIPS ↓
Random cluster selection	16.21	0.690	0.4980
Fixed prompt (“object”) + w/o CLIP	16.74	0.708	0.4610
Fixed prompt + w/CLIP verification	17.18	0.728	0.4310
LLM prompt + w/o CLIP verification	17.39	0.738	0.4180
**Full SAGS (Ours)**	**17.82**	**0.754**	**0.3950**

*Note:* SAGS denotes Semantic-Aware Gaussian Selector; LLM denotes large language model; and CLIP denotes contrastive language–image pre-training. “w/” and “w/o” denote “with” and “without”, respectively. PSNR denotes peak signal-to-noise ratio; SSIM denotes structural similarity index measure; and LPIPS denotes learned perceptual image patch similarity. ↑ and ↓ indicate that higher and lower values are preferred, respectively, rather than specifying a row-sorting order.

**Table 8:** **Effect of the Gaussian initialization strategy on inpainting quality.** The best results are shown in bold.

Initialization Strategy	PSNR ↑	SSIM ↑	LPIPS ↓
Random Sampling	16.39	0.695	0.4870
Boundary Copy Only	17.04	0.722	0.4360
**Ours (Boundary + Uniform)**	**17.82**	**0.754**	**0.3950**

*Note:* PSNR denotes peak signal-to-noise ratio; SSIM denotes structural similarity index measure; and LPIPS denotes learned perceptual image patch similarity. ↑ indicates that a higher value is better, whereas ↓ indicates that a lower value is better; the arrows do not indicate the ordering of the initialization strategies.

**Table 9:** **Hyperparameter sensitivity analysis.** The default settings are shown in bold and provide the best overall balance among the evaluated metrics.

Hyperparameter	Value	PSNR ↑	SSIM ↑	LPIPS ↓
CLIP Threshold $\tau_{clip}$	0.20	17.41	0.741	0.4180
	**0.25**	**17.82**	**0.754**	**0.3950**
	0.30	17.58	0.746	0.4090
DBSCAN ϵ	0.05	17.33	0.737	0.4230
	**0.10**	**17.82**	**0.754**	**0.3950**
	0.15	17.52	0.744	0.4110
Virtual Views $N_{virt}$	8	17.49	0.743	0.4120
	**12**	**17.82**	**0.754**	**0.3950**
	16	17.78	0.752	0.3970

*Note:* CLIP denotes contrastive language–image pre-training; DBSCAN denotes density-based spatial clustering of applications with noise; and $N_{\mathrm{virt}}$ denotes the number of virtual views. PSNR denotes peak signal-to-noise ratio; SSIM denotes structural similarity index measure; and LPIPS denotes learned perceptual image patch similarity. ↑ and ↓ indicate that higher and lower values are preferred, respectively, rather than specifying the ordering of the hyperparameter settings.

## Data Availability

The IMFine, SPIn-NeRF, and Inpaint360GS datasets analyzed in this study are publicly available from their respective official project repositories cited in the manuscript. The source code, configuration files, and evaluation scripts are available from the anonymous repository at https://github.com/OuYangFengOyf/OVR-GS (accessed on 16 July 2026).
